# Multiphysics modelling of photon, mass and heat transfer in coral microenvironments

**DOI:** 10.1098/rsif.2021.0532

**Published:** 2021-09-01

**Authors:** Shannara Kayleigh Taylor Parkins, Swathi Murthy, Cristian Picioreanu, Michael Kühl

**Affiliations:** ^1^ Department of Biotechnology, Delft University of Technology, Van der Maasweg 9, 2629 HZ Delft, The Netherlands; ^2^ The Novo Nordisk Foundation Center for Biosustainability, Technical University of Denmark, Kemitorvet 220, 2800 Kgs. Lyngby, Denmark; ^3^ Marine Biology Section, Department of Biology, University of Copenhagen, Strandpromenaden 5, 3000 Helsingør, Denmark; ^4^ Biological and Environmental Sciences and Engineering Division, Water Desalination and Reuse Center, King Abdullah University of Science and Technology, Thuwal 23955-6900, Saudi Arabia; ^5^ Climate Change Cluster, University of Technology Sydney, Ultimo, New South Wales 2007, Australia

**Keywords:** modelling, light, radiative transfer, temperature, symbiosis

## Abstract

Coral reefs are constructed by calcifying coral animals that engage in a symbiosis with dinoflagellate microalgae harboured in their tissue. The symbiosis takes place in the presence of steep and dynamic gradients of light, temperature and chemical species that are affected by the structural and optical properties of the coral and their interaction with incident irradiance and water flow. Microenvironmental analyses have enabled quantification of such gradients and bulk coral tissue and skeleton optical properties, but the multi-layered nature of corals and its implications for the optical, thermal and chemical microenvironment remains to be studied in more detail. Here, we present a multiphysics modelling approach, where three-dimensional Monte Carlo simulations of the light field in a simple coral slab morphology with multiple tissue layers were used as input for modelling the heat dissipation and photosynthetic oxygen production driven by photon absorption. By coupling photon, heat and mass transfer, the model predicts light, temperature and O_2_ gradients in the coral tissue and skeleton, under environmental conditions simulating, for example, tissue contraction/expansion, symbiont loss via coral bleaching or different distributions of coral host pigments. The model reveals basic structure–function mechanisms that shape the microenvironment and ecophysiology of the coral symbiosis in response to environmental change.

## 1. Introduction

The symbiosis between calcifying coral animals and photosynthetic microalgae drives the formation of the very foundation of coral reefs, one of the most diverse marine ecosystems that provide important services for fisheries, coastal protection and tourism. Endosymbiotic dinoflagellates in the family Symbiodiniaceae [1] are located in the coral host endoderm cells and provide the heterotrophic coral animal with carbohydrates and O_2_ via their photosynthesis. In return, the coral host provides its symbionts with essential nutrients and a safe environment in the thin coral tissue, which can range in thickness from less than 0.1 to several millimetres [2]. Light is the driving force of the coral symbiosis [3], but excess light levels in combination with abnormal temperatures of the surrounding seawater can trigger the degradation or expulsion of symbionts, leaving the discoloured coral animal tissue and its underlying, white carbonate skeleton behind as a result of the so-called coral bleaching [4]. Increasing seawater temperature as a result of ongoing climate change is currently accelerating the occurrence of mass bleaching events that have dire consequences for coral reefs worldwide [5,6]. Thus, understanding how environmental stress affects the photobiology and ecophysiology of corals has increased importance, with a special focus on the *in vivo* light environment driving the coral–algal symbiosis.

Corals inhabit a wide range of reef habitats ranging from the shallow reef flat under highly dynamic flow, temperature and light conditions (including high UV levels), down to mesophotic depths of 30 to more than 100 m off the reef crest under more stable flow and temperature conditions and increasing light limitation [3,7]. The morphological and behavioural plasticity of the coral host and the composition of its associated photosymbionts and microbiome thus represents a highly adapted holobiont that can operate efficiently under varying conditions of solar irradiance [8]. Yet the underlying optical properties and mechanisms that govern such adaptation and link the structure–function interactions in corals remain underexplored.

Detailed experimental studies of coral thermal microenvironments have demonstrated solar irradiance-driven heating of coral tissue and skeleton in the presence of a thick thermal boundary layer (TBL; 2.6 mm) [9,10], especially at low water flow (0.2–0.6 cm s^−1^) and high irradiance levels [11,12]. Both the concentration boundary layer (DBL; the diffusion-dominated water layer adjacent to the coral surface) that strongly regulates solute exchange in corals [13,14] and the TBL are affected by the interaction between the surrounding seawater and the coral colony structure. Consistently thinner DBL and TBL develop at higher flow velocity and over smoother coral surfaces [11].

Coral light absorption drives both accumulation of O_2_ via efficient symbiont photosynthesis and local heating [15,16]. It is thus important to understand light harvesting and propagation, as well as the underlying inherent optical parameters of the coral tissue and skeleton. The use of optical microsensors [17,18] has revealed pronounced light gradients in thick-tissue corals [19] that can support symbionts with different photoacclimation in different tissue depths [20,21]. The coral tissue light field is strongly affected by scattered light from the coral skeleton [22] and different tissue components [16,23–25], which affects photon path length and can lead to local light enhancement for coral photosynthesis.

Estimates of the inherent scattering and absorption properties of the living coral skeleton and tissue (i.e. absorption and scattering coefficients and scattering anisotropy) have only recently become available using techniques developed in biomedical optics [24–26]. Based on such measurements or assumptions of inherent optical properties, simple probabilistic models of light propagation in corals have been developed [27,28], assuming a simple two-layer configuration of coral tissue and skeleton and employing Monte Carlo (MC) modelling of radiative transfer in such structures. However, corals consist of a mucus-covered multi-layered tissue with a water-filled gastric cavity, and all these compartments can potentially affect light propagation because of refractive index mismatches [23]. The individual layers are very thin, and it is presently not possible to align microscale measurements of light or extraction of optical properties to individual coral tissue layers owing to a lack of spatial resolution (typically 50–100 µm) and possible disturbances of tissue integrity and boundary layers during measurements. This is also true for microscale measurements of temperature and key chemical parameters involved in coral metabolism such as O_2_, CO_2_ and pH. Our understanding of how coral tissue stratification and the underlying carbonate skeleton affect internal gradients of light, temperature and chemical species, and thereby the overall photon, heat and mass transfer in the coral holobiont, thus remains incomplete.

The interaction of corals with water flow and irradiance and the resulting thermal microenvironments of corals over complex colony morphologies have been explored by, for example, Ong *et al*. [29–32], who employed three-dimensional (3D) computational fluid dynamics modelling in combination with optical ray tracing for a range of coral colony morphologies. Several other studies have focused on the impact of complex coral morphology and flow patterns on heat and mass transfer at the coral surface, where the roughness of the coral surface played a large role [33–36]. However, no attempts have been made to link the light distribution to heat and mass transfer at a more detailed tissue scale, including the effect of intra-tissue stratification and interactions with the underlying coral skeleton.

**Figure 1.**
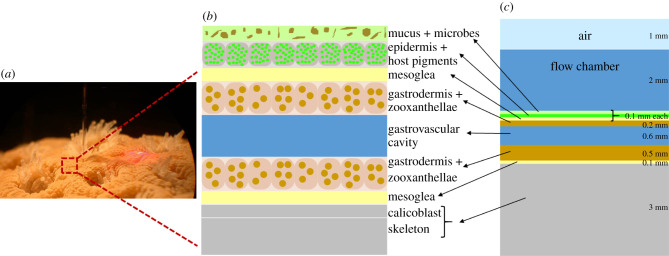
Schematic illustration of tissue organization in a coral. A coral colony (*a*) is composed of clonal polyps with connective tissue, on top of a joint calcium carbonate skeleton. The coral tissue has a simple organization (*b*) composed of an external epidermis (ectoderm) and an endoderm (gastrodermis) separated by a gelatinous, fibrous mesoglea layer. The endoderm harbours dinoflagellate microalgae as endosymbionts (zooxanthellae). The water-filled gastric cavity separates upper and lower tissue, where the upper tissue ectoderm is typically covered by a mucus layer, and the lower tissue ectoderm is modified into a calicoblast layer, wherein the formation of the coral skeleton is initiated. This basic tissue organization was included in the coral slab model (*c*), which consists of external media (air, seawater), upper tissue compartment, the gastric cavity, the lower tissue compartment and the coral skeleton, including the calicoblast layer. The thickness of each layer is included and also available in table 1.

In this study, we developed a computational model linking light, heat and mass transfer in a simple coral slab geometry, taking all coral tissue layers and the coral skeleton into account (figure 1). Based on 3D MC simulations of light propagation using different optical properties for every layer, the resulting light field was coupled with heat transfer and metabolism (photosynthesis and respiration) inside the coral to numerically simulate temperature and O_2_ concentration profiles for different coral configurations. This enabled us to (i) predict the effect of varying coral host pigment density, tissue contraction and expansion, mucus production and symbiont loss under coral bleaching on light, temperature and O_2_ gradients across coral tissue and skeleton and (ii) check the model predictions with existing experimental data in the literature. We note that our approach does not aim to represent the full complexity of flow and light interactions with 3D coral structures over large spatial scales and in the natural habitat. However, the multiphysics model can be used as an exploratory tool for predicting changes in coral holobiont microenvironments under various environmental conditions at the local (i.e. sub-millimetre) tissue scale under conditions frequently applied in microsensor analyses of corals in flow chambers.

## 2. Model description

We developed a multiphysics computational approach combining MC modelling of radiative transfer with numerical modelling of radiation-driven heating and O_2_ production in a coral slab (including layers of air, water, mucus, several coral tissue layers and coral skeleton). The model combines coral optics with the laminar flow of water, heat transfer and mass transport of O_2_ coupled with photosynthesis and respiration reactions. By varying optical, geometrical and structural parameters of the coral model, the resulting profiles of light, temperature and O_2_ across the different coral compartments provided insight into the role of coral host pigments, tissue contraction and expansion, mucus production under stress conditions and symbiont loss during coral bleaching on the physico-chemical microenvironment of coral tissue and skeleton.

### 2.1. Model geometry

**Figure 2.**
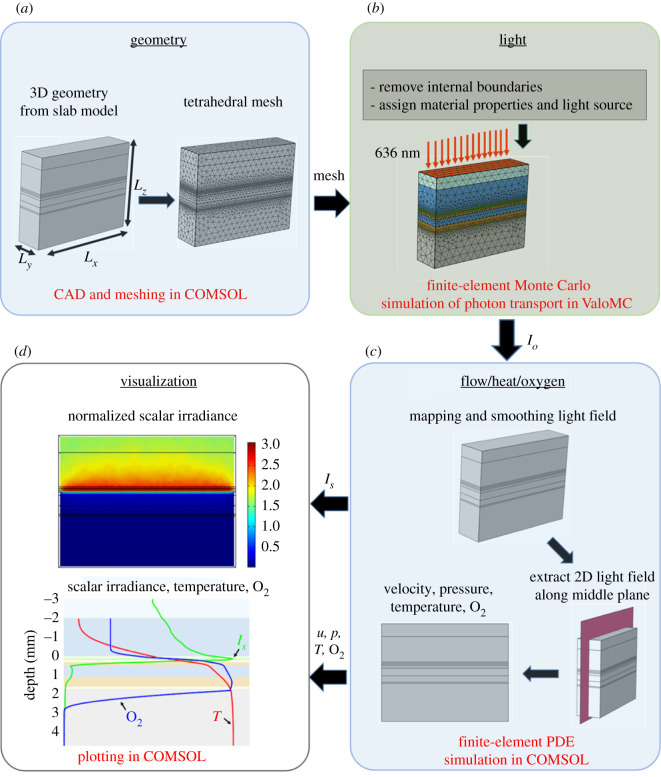
Schematic showing the different steps involved in the multiphysics simulation of radiative, heat and mass transfer in the coral slab model: (*a*) model design, (*b*) finite-element MC simulation of photon propagation (light), (*c*) finite-element PDE simulation of flow, heat and mass transfer and (*d*) visualization of results. Model boundary conditions are shown in electronic supplementary material, figure S1.

The model geometry represents a coral slab with layers of air, water, mucus, coral tissue and coral skeleton (figure 2). A 3D model was initially constructed, with the goal of representing any coral geometry. The simulation of a hemispherical coral immersed in a parallelepiped volume of liquid is presented here only as an illustration of the approach. However, since, at the studied scale (millimetres), the tissue layers appear quasi-planar, simpler and computationally more efficient, one-dimensional (1D) and two-dimensional (2D) geometry reductions were also proposed.

The 1D model would represent the gradients over the coral tissue depth, but not describe directly the formation of mass/heat transfer boundary layers in the water film adjacent to the coral surface (i.e. a DBL/TBL thickness would have to be imposed rather than computed). While keeping the layered structure, we opted therefore for a 3D geometry in the computation of the light field, which supplied input to a 2D model for the heat and mass transfer calculations (with total height or thickness *L_z_* and width *L_x_*). The 3D slab model of the coral was constructed in COMSOL Multiphysics (v. 5.4; COMSOL Inc., Burlington, MA, USA) by extending the 2D slab in the length by *L*_*y*_.

Fine-scale measurements (e.g. with microsensors) of coral microenvironments and ecophysiology have mostly been done in flow chambers (e.g. [15]), and only a few *in situ* measurements have been published (e.g. [10,37]). Thus, the model parameters were chosen to mimic such flow chamber measurements, i.e. stationary laminar, unidirectional flow, constant temperature and moderate incident downwelling irradiance. An air layer was included because the light source during experiments would be located above the water, thus the refractive index mismatch between air and water had to be considered in the model. The water layer is the only medium flowing and was included to represent the temperature and O_2_ concentration profiles in the thermal and mass transfer boundary layers that are usually measured. The coral tissue comprises several functional layers from water to the skeleton surface [23]: mucus, epidermis (sometimes containing fluorescent or non-fluorescent coral host pigments [38]), oral mesoglea, gastrodermis (with photosynthetic symbionts), a water-filled gastrovascular cavity, aboral gastrodermis (also with symbionts) and aboral mesoglea (figure 2, slab model). The thickness of the different coral layers, *L_z,i_* (table 1), used in our model, followed experimental measurements reported in the literature on thick-tissue faviid corals, where the effect of variable tissue thicknesses was also investigated in several studies. The skeleton and calicoblast were combined, since both layers were assumed to be dominated by porous aragonite (CaCO_3_). The width of the 2D slab, *L_x_*, was chosen large enough to establish the thermal and diffusive boundary layers, while still allowing a reasonable computational time for the MC simulations of the light field.

**Table 1.** Model parameters for coral geometry and optics.

parameter	symbol	value	unit	source
*geometry*				
length slab	*L_x_*	10	mm	chosen
width slab	*L_y_*	2.5	mm	chosen
thickness slab layers				
air	*L*_*z*,air_	1	mm	[28]
water	*L*_*z*,wat_	2	mm	[28]
mucus	*L*_*z*,muc_	0.1	mm	[20]
epidermis	*L*_*z*,ep_	0.1	mm	[16]
mesoglea	*L*_*z*,mes_	0.1	mm	[20]
oral gastrodermis	*L*_*z*,og_	0.2	mm	[20]
gastrovascular cavity	*L*_*z*,gas_	0.6	mm	[20]
aboral gastrodermis	*L*_*z*,ag_	0.5	mm	[20]
skeleton	*L*_*z*,skel_	3	mm	[28]
*optics*				
absorption coefficients				
air	*μ*_*a*,air_	1 × 10^−4^	cm^−1^	[39]
water	*μ*_*a*,wat_	3.6 × 10^−3^	cm^−1^	[39]
mucus	*μ*_*a*,muc_	3.6 × 10^−3^	cm^−1^	[39]
epidermis	*μ*_*a*,ep_	3.6 × 10^−3^	cm^−1^	[39]
mesoglea	*μ*_*a*,mes_	1.6 × 10^−3^	cm^−1^	[40]
oral gastrodermis	*μ*_*a*,og_	75.95	cm^−1^	[27]
gastrovascular cavity	*μ*_*a*,gas_	3.6 × 10^−3^	cm^−1^	[39]
aboral gastrodermis	*μ*_*a*,ag_	30.1	cm^−1^	[27]
skeleton	*μ*_*a*,skel_	0.01	cm^−1^	[28]
scattering coefficients				
air	*μ*_*s*,air_	1 × 10^−5^	cm^−1^	[39]
water	*μ*_*s*,wat_	1 × 10^−5^	cm^−1^	[39]
mucus	*μ*_*s*,muc_	1.15	cm^−1^	[41]
epidermis	*μ*_*s*,ep_	126	cm^−1^	[24]
mesoglea	*μ*_*s*,mes_	150	cm^−1^	[40]
oral gastrodermis	*μ*_*s*,og_	97.65	cm^−1^	[27]
gastrovascular cavity	*μ*_*s*,gas_	1 × 10^−5^	cm^−1^	[39]
aboral gastrodermis	*μ*_*s*,ag_	38.7	cm^−1^	[27]
skeleton	*μ*_*s*,skel_	34	cm^−1^	[28]
anisotropy coefficients				
air	*g*_air_	1	—	[39]
water	*g*_wat_	1	—	[39]
mucus	*g*_muc_	0.93	—	[41]
epidermis	*g*_ep_	0.34	—	[24]
mesoglea	*g*_mes_	0.68	—	[40]
oral gastrodermis	*g*_og_	0.98	—	[27]
gastrovascular cavity	*g*_gas_	1	—	[39]
aboral gastrodermis	*g*_ag_	0.98	—	[27]
skeleton	*g*_skel_	0.9	—	[28]
refractive indices				
air	*n*_air_	1	—	[39]
water	*n*_wat_	1.33	—	[39]
mucus	*n*_muc_	1.36	—	[41]
epidermis	*n*_ep_	1.38	—	[28]
mesoglea	*n*_mes_	1.45	—	[42]
oral gastrodermis	*n*_og_	1.42	—	[43]
gastrovascular cavity	*n*_gas_	1.33	—	[39]
aboral gastrodermis	*n*_ag_	1.42	—	[43]
skeleton	*n*_skel_	1.66	—	[44]
fraction absorbed light to heat	*f*_heat_	0.96	—	[15]
fraction absorbed light to photosynthesis	*f*_ps_	0.04	—	[15]

### 2.2. Model solution

The steps taken in the model definition and solution are schematically presented in figure 2. A schematic of the coral slab model with all the boundary conditions for light, flow, heat and mass transfer simulation is shown in the electronic supplementary material, figure S1 together with a detailed description of the model physics and equations used. The light field in the 3D slab model was computed by MC simulations using the ValoMC toolbox [45] in Matlab (MathWorks, Natick, MA, USA). The resulting light field was smoothed on a finite-element tetrahedral mesh (overall maximum element size 0.55 mm) using a weak form of partial differential equation (PDE) in COMSOL Multiphysics. A mesh-independence study in the base-case scenario showed that a minimum element size of 2 µm was sufficient for the MC light simulations (electronic supplementary material, figure S2). A slice from the smoothed 3D light field was extracted through the middle of the domain and used in the calculations of the 2D water flow, heat transfer and O_2_ transport with reactions. These 2D PDEs were solved on a square mesh also in COMSOL Multiphysics (overall maximum element size 0.1 mm). For the solution of oxygen concentration and temperature profiles, a mesh size below 20 µm made no discernable change in the profiles, so we used a 10 µm mesh size along with the tissue depth. Finally, the 2D fields or 1D profiles of scalar irradiance, temperature and dissolved O_2_ concentration were visualized within COMSOL.

### 2.3. Model parameters

All model parameters are listed in tables 1 and 2. The thickness of the different coral layers was taken from published experimental studies; however, the effect of variable thicknesses was also investigated in the present study. The optical properties were partly based on values in faviid corals (such as *Favia* sp.). The incident downwelling photon irradiance of 320 µmol photon m^−2^ s^−1^ used in most simulations is representative of typical irradiance values saturating photosynthesis in shallow water coral without inducing photoinhibition. Several assumptions were made to obtain optical properties for every tissue layer: (i) the mucus layer was assumed to have a 10–20% sugar content in water [57], (ii) the coral host pigments were assumed to be only located in the epidermis with no light absorption at 636 nm [58], (iii) the mesoglea was assumed to have a 10–20% lipid content in water [59], (iv) the symbionts were only located in the oral and aboral gastrodermis, and (v) the gastrovascular cavity was assumed to be pure water [23].

**Table 2.** Model parameters for flow, heat, mass transport and biokinetics.

parameter	symbol	value	unit	source
*flow and heat*				
viscosity water	*η*_wat_	0.0009	Pa s	[46]
densities				
water	*ρ*_wat_	997	kg m^−3^	[46]
tissue (all)	*ρ*_tis_	1109	kg m^−3^	[47]
skeleton	*ρ*_skel_	2930	kg m^−3^	[48]
heat conductivities				
water	*k*_wat_	0.6	W m^−1^ K^−1^	[46]
tissue (all)	*k*_tis_	0.37	W m^−1^ K^−1^	[47]
skeleton	*k*_skel_	1.5	W m^−1^ K^−1^	[9]
heat capacities				
water	*C*_*p*,wat_	4183	J kg^−1^ K^−1^	[46]
tissue (all)	*C*_*p*,tis_	3391	J kg^−1^ K^−1^	[47]
skeleton	*C*_*p*,skel_	811	J kg^−1^ K^−1^	[49]
*mass transport*				
diffusion coefficients				
O_2_ in water	*D*_O_2_,_wat__	2 × 10^−9^	m^2^ s^−1^	[50]
O_2_ in tissue (all)	*D*_O_2_,_tis__	1 × 10^−9^	m^2^ s^−1^	[51]
O_2_ in skeleton	*D*_O_2_,_skel__	2 × 10^−11^	m^2^ s^−1^	from water, assumed 1% porosity
*biokinetics*				
symbiont light respiration rate	*R*_rp,sym_	1.2 × 10^−2^	mol m^−3^ s^−1^	[52]
density symbionts in tissue	*ρ*_sym,tis_	2.7 × 10^6^	cells cm^−2^	[53]
quantum efficiency oral gastrodermis	*Q*_og_	0.060	mol mol^−1^	[15]
quantum efficiency aboral gastrodermis	*Q*_ag_	0.102	mol mol^−1^	[15]
*P/R* ratio in gastrodermis	*PR*	3.5	—	[54]
O_2_ half-saturation coefficient	*K*_O_2__	3.13 × 10^−3^	mol m^−3^	typical value of 0.1 mg l^−1^
O_2_ consumption rate skeleton	*R*_cp,skel_	3.75 × 10^−5^	mol m^−3^ s^−1^	[55]
*experimental parameters*				
average water velocity	*v*_*0*_	0.4	cm s^−1^	[15]
ambient temperature	*T*_*0*_	25	°C	[15]
inlet O_2_ concentration	*c*_*0*,O_2__	0.25	mol m^−3^	[56]
incident downwelling photon irradiance	*I*_id_	320	µmol photon m^−2^ s^−1^	[15]

## 3. Results and discussion

**Figure 3.**
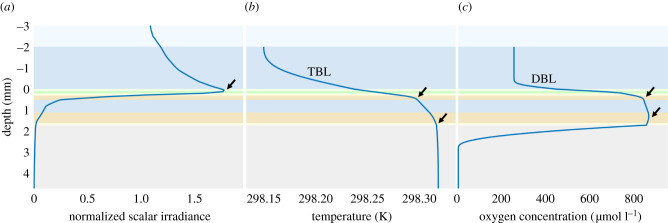
Depth profiles of normalized scalar irradiance (*a*), temperature (*b*) and O_2_ concentration (*c*) as simulated by the coral slab model for the baseline case (all parameters as in table 1). The arrow in the normalized scalar irradiance depth profile indicates a strong light enhancement by refractive index mismatches and the presence of scattering coral host pigments in the epidermis. The TBL and DBL are visible above the coral tissue surface in the temperature and O_2_ concentration depth profiles. The arrows in the temperature and O_2_ concentration depth profiles indicate peaks in the temperature and O_2_ concentrations, which are due to the high light absorption in the oral (upper arrow) and aboral (lower arrow) gastrodermis resulting in strong local heat dissipation and high photosynthesis rates, respectively.

To gain more insight into coral optics and its effects on *in vivo* temperature and O_2_ concentration, simulations were performed with the 2D coral slab model. Five scenarios were simulated to investigate several mechanisms of coral photoacclimation, including the role of coral host pigments, tissue movement, mucus production and bleaching. The results of all scenarios have multiple features in common, which are explicitly shown for the baseline case (figure 3; all parameters as in table 1).

(i) All depth profiles of normalized scalar irradiance show a strong enhancement of light at the coral tissue surface due to scattering, caused by the refractive index mismatches in the tissue and, for example, the presence of highly scattering coral host pigments, which corresponds with the results of experimental light measurements [16,37,60]. Overall, the light was strongly attenuated through the coral tissue, also corresponding with experimental studies on thick-tissue corals [19,20].
(ii) The temperature of the coral tissue surface in light was always higher than the ambient water temperature (except for the completely bleached coral case), which is in line with temperature microsensor measurements in several experimental studies [9,12]. However, the results of the simulated scenarios suggest that the *in vivo* temperature at the site of the Symbiodiniaceae cells is even higher than the coral tissue surface temperature.

So far it has not been possible to perform microscale temperature measurements at sufficient resolution to confirm the predicted temperature profiles in the coral tissue. Experimentally, the temperature has been measured with up to 0.05–0.10°C precision [9–12], though only in the liquid towards the coral surface and not within the coral tissue. As expected, the temperature profile peaked in the oral and aboral gastrodermis owing to their relatively high absorption coefficient resulting in a strong local heat dissipation. The temperature stayed almost constant below the aboral gastrodermis and into the skeleton, which acted as a thermal isolator because of lower heat conduction properties. The maximum temperature difference was generally around 0.1°C, which is a result of the relatively low incident downwelling irradiance (320 µmol photon m^−2^ s^−1^) in most of our simulations. For shallow coral reefs, the incident downwelling irradiance (400–700 nm) can reach up to 2400 µmol photon m^−2^ s^−1^ [9,37]. Therefore, when we ran simulations with higher incident downwelling irradiance values, the model predicted maximum temperature differences of up to 1°C (electronic supplementary material, figure S3), which is in line with experimental laboratory measurements [9,12] and *in situ* observations on a coral reef flat under calm conditions [10]. In a real-world scenario, temperature dynamics on coral reefs can certainly be more variable in time and space, e.g. because of weather and seasons, tidal currents, wave actions and interactions of turbulent flow with the 3D topography of corals [32–36]. However, a detailed discussion of such interactions is beyond the scope of this study.

(iii) The depth profiles of O_2_ concentration in light all exhibited a quasi-parabolic shape in the tissue layer (except for the completely bleached coral case). This is indicative of net photosynthetic O_2_ production, as shown in multiple experimental studies [13,15]. The O_2_ concentration also peaked in the oral and aboral gastrodermis, because the Symbiodiniaceae cells in these layers absorb the light for photosynthesis resulting in local O_2_ production. Furthermore, the depth profiles of O_2_ concentration showed how the low diffusivity of the coral skeleton resulted in O_2_ accumulation at the skeleton surface, again confirming experimental observations [19].
(iv) Overall, the TBL and DBL thicknesses estimated from all simulated depth profiles of temperature and O_2_ concentration were within the range measured in experiments [11]. We note, however, that, while providing a very good qualitative match to experimental data, a detailed quantitative comparison between the simulated results and experiments is not justified because the results depend on the properties of specific corals. Here, we have focused on thick-tissue faviid corals with the epidermal location of coral host pigments, but corals exhibit a large morphological plasticity in overall colony morphology and tissue organization, composition and thickness [61]. Thus, only a qualitative comparison with published experimental data is presented here for all modelled scenarios, as it is not possible to quantitatively compare the simulated thickness of the TBL and DBL with experimental measurements, where the boundary layer thicknesses are highly dependent on the flow velocity and coral morphology [11]. In the following, we use our coral slab model to explore the effect of changing tissue optical properties on the light field, temperature and O_2_ microenvironment in thick-tissue corals and compare model predictions with experimental evidence in the literature.

### 3.1. Granular distribution of coral host pigments provides more shielding of Symbiodiniaceae

Corals can have their fluorescent or non-fluorescent host pigments arranged in tightly packed granules or as a more diffuse distribution in their tissues, and some corals can even modulate their host pigment density via tissue contraction/expansion [61]. It was recently shown that host pigments can significantly affect light scattering and thereby light absorption and heat dissipation in coral tissue [16]. To further explore the role of differently distributed host pigments in tissue, we implemented the hitherto only published estimates on the *in vivo* optical properties of coral host pigments in the model, where the anisotropy factor of the epidermis (*g*_ep_) is 0.34 (nearly isotropic scattering) or 0.96 (mostly forward scattering) for granular or diffuse fluorescent proteins, respectively [24].

**Figure 4.**
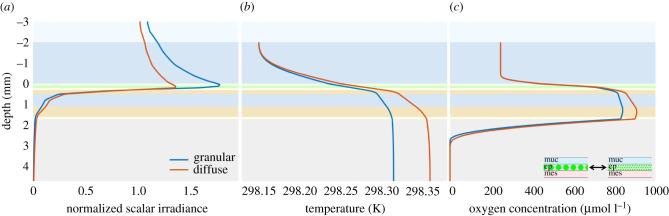
Simulated depth profiles of normalized scalar irradiance (*a*), temperature (*b*) and O_2_ concentration (*c*) in corals with granular versus diffuse distribution of coral host pigments in the epidermis.

Model results revealed a noticeable difference in the physico-chemical tissue microenvironment between corals with a granular and a diffuse distribution of host pigments (figure 4), pointing to an important role of host pigments in modulating coral metabolism. A granular distribution of host pigments caused a significantly higher light enhancement at the coral tissue surface and less efficient light penetration to deeper tissue layers than in corals with a more diffuse distribution of host pigments. Such increased light enhancement at the coral tissue surface and more rapid vertical light attenuation was indeed found to be characteristic for coral tissues with a granular distribution of host pigments [16,24].

Another model prediction was that tissue heating and O_2_ concentration in deeper tissue layers reached lower values in corals with a granular distribution of host pigments, providing more shielding of the underlying tissue, especially of the oral and aboral gastrodermis with Symbiodiniaceae cells, as compared to corals with a diffuse distribution of fluorescent proteins. Such a mechanism was originally hypothesized by Lyndby *et al*. [16].

### 3.2. Higher densities of coral host pigments enhance shielding of underlying tissue

**Figure 5.**
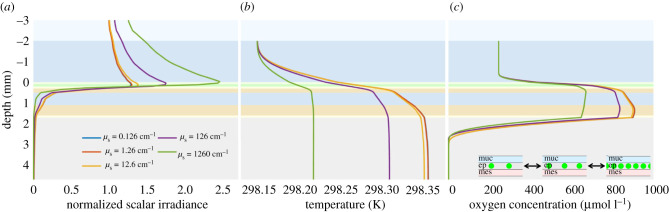
Simulated depth profiles of normalized scalar irradiance (*a*), temperature (*b*) and O_2_ concentration (*c*) in corals with different densities of granular distributed host pigments (here simulated via different scattering properties in the epidermal layer).

The density of host pigments in coral tissue differs between corals based on species or location [38,62] or between contracted and relaxed coral tissue [16,61,63]. We explored the effect of different host pigment densities on the coral microenvironment by varying the scattering coefficient (mainly linked to the presence of host pigments) in the epidermis (*µ*_*s*,ep_) over a range typical for corals (0.126 cm^−1^, 1.26 cm^−1^, 12.6 cm^−1^, 126 cm^−1^ and 1260 cm^−1^) [26]. Our model calculations predicted significant changes in the depth profiles of normalized scalar irradiance, temperature and O_2_ concentration between low and high densities of granular host pigments in the epidermis (figure 5). When the scenario was changed to a more diffuse distribution of host pigments, the same results were obtained, but with overall lower shielding effects (electronic supplementary material, figure S4). Higher host pigment densities increased the light enhancement at the coral tissue surface, shielding the underlying tissue layers from excess light. With the increased shielding, higher host pigment densities resulted in lower coral tissue temperature and lower O_2_ concentrations owing to less heat dissipation and photosynthesis in deeper layers. These simulation results are mostly in line with the hypothesized mechanism for concentrating coral host pigments by tissue contraction, which was based on experimental observations by Lyndby *et al*. [16]. However, this mechanism also implies that the coral tissue surface temperature increases with increasing host pigment density up to a saturating pigment density. The simulated depth profiles of temperature did not reflect this, probably because of the localized light enhancement in a region of low light absorption (the mucus, epidermis and oral mesoglea all have relatively low absorption coefficients). Thus, the simulated results only suggest enhanced shielding of underlying tissue layers with higher host pigment densities.

The proposed photoprotective function of coral host pigments suggested from experiments [38,62] is completely supported by the results from our simulated scenario. Increased shielding by higher host pigment densities is found in shallow water corals that experience significantly higher irradiance and contain higher densities of fluorescent proteins than corals located deeper on the reefs, i.e. mesophotic corals [38,62]. Furthermore, corals with higher host pigment densities are more resilient to coral bleaching [4,38] as increased shielding can lead to less heating of the coral and lower chances of photoinhibition of the Symbiodiniaceae cells *in hospite*, and we thus speculate that corals with granular instead of diffuse distribution of host pigments might be more resilient to bleaching.

### 3.3. Tissue contraction and expansion alters the *in vivo* light field in corals

Corals are able to move (contract or expand) their tissue and thus the tissue thickness, e.g. in response to light changes [23,64]. We simulated the effects of tissue contraction and expansion on the coral microenvironment by varying total tissue thickness between 1.7 mm and 0.85 mm, respectively. This simplified scenario assumes only sideways expansion, which means the total amounts of host pigments in the epidermis and Symbiodiniaceae cells in the oral and aboral gastrodermis are halved in the expanded compared with the contracted state.

**Figure 6.**
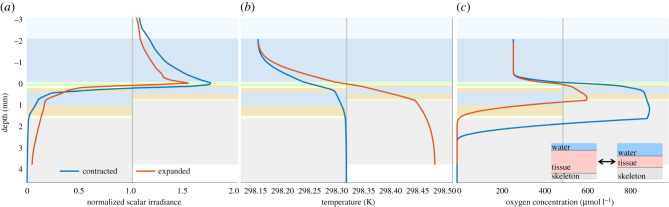
Simulated depth profiles of normalized scalar irradiance (*a*), temperature (*b*) and O_2_ concentration (*c*) in a coral with contracted (left side in each panel) and sideways-expanded (right side in each panel) tissue.

All simulated depth profiles showed a drastic difference between the contracted and expanded state of the coral tissue (figure 6). The contraction of the coral tissue resulted in higher light enhancement at the coral tissue surface, while expansion resulted in more light reaching deeper in the coral tissue, which is in line with results from experiments [23] and underscores that tissue contraction upon high light conditions can be a protective mechanism of the coral.

Interestingly, the simulations indicated that the oral tissue layers experience higher light levels in the contracted state, while the aboral tissue layers experience higher light levels in the expanded state. We speculate that this may be a niche shaping factor enabling differential light acclimatization between Symbiodiniaceae residing in the oral and aboral gastrodermis, as, for example, observed in thick-tissue corals [20]. With more light reaching the skeleton, the coral tissue reaches a higher temperature in the expanded state. Lastly, the O_2_ concentration is higher in the contracted state because of the increased density of Symbiodiniaceae cells. To confirm these predictions of light, temperature and O_2_ concentration during tissue movements, further research is required.

An analogous scenario to the comparison between the contracted and expanded state of coral tissue is the comparison between thick- and thin-tissue corals. This simplified analogue is justified, since thin-tissue corals generally have lower densities of host pigment [4] and Symbiodiniaceae cells [53]. In experiments, thick-tissue (massive) corals reached higher tissue surface temperatures than thin-tissue (branching) corals [9,12], while our simulations suggest the opposite. However, this difference between experiments and simulations could be caused by the assumed optical and thermal properties of the coral skeleton that remain poorly resolved across different coral species.

The skeleton of thick-tissue (massive) and thin-tissue (branching) corals can be rather different in terms of structure, porosity and scattering properties [65,66], resulting in different tissue heating properties [9,12]. Thus, more detailed studies, especially of skeletal heat transfer, remain to be done. The skeleton properties were kept constant in all our simulations, thus not capturing the important differences in the skeleton of two types of corals. Furthermore, the coral slab model does not include the overall 3D structure of the coral, where the difference in morphology between massive and branching corals also affects heat transfer and the temperature at the coral tissue surface significantly [9].

More complex 3D models including these coral structural features will rely on better characterization of the thermal (e.g. heat capacity and conductivity) and optical (scattering and absorption) properties of coral tissue and skeleton, as well as additional experiments quantifying the differences in *in vivo* light fields, temperature and O_2_ dynamics between the thick- and thin-tissue corals.

### 3.4. Increased mucus production can enhance O_2_ accumulation within the coral tissue

All corals produce mucus, which has multiple functions, e.g. to clean their tissue surface and hosting an important part of the coral microbiome [67,68]. Under environmental stress, corals increase their mucus production significantly, e.g. as a result of increased ambient temperature, exposure to air or excess UV radiation [3,57]. The role of increased mucus production for the coral microenvironment, and especially its effects on the *in vivo* light field, temperature and O_2_ concentration, remains largely unexplored, thus simulations were performed to investigate this function. The thickness of the mucus (*z*_muc_) was changed to cover a range of mucus production values typical for corals (0.05 mm, 0.1 mm and 0.15 mm) [69].

**Figure 7.**
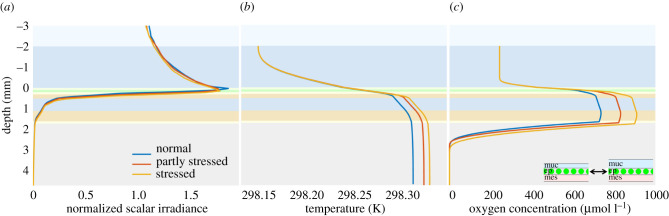
Simulated depth profiles of normalized scalar irradiance (*a*), temperature (*b*) and O_2_ concentration (*c*) in the coral slab model, for increased mucus production under environmental stress.

Our simulations showed only a slight enhancement of normalized scalar irradiance and temperature in the coral tissue with increased mucus production, while a much larger effect was predicted on the depth profile of O_2_ concentration (figure 7). The slightly deeper light penetration with increased mucus production was probably caused by enhanced internal reflection due to the refractive index mismatch at the mucus–water interface. A thicker mucus layer enlarges the region available for photon trapping and thus leads to enhanced internal reflection. The slight increase in coral tissue temperature results from the slightly deeper light penetration and thus radiative heat dissipation. The relatively large impact of increased mucus production on the O_2_ concentration can be explained by the combined effect of the deeper light penetration stimulating Symbiodiniaceae photosynthesis in the tissue and a larger diffusion barrier for gas exchange between tissue and overlying water caused by the thicker mucus layer. To our knowledge, the microenvironmental consequences of increased mucus production in corals remain unexplored, and experimental verification is required to confirm these model predictions of the function and mechanism of increased mucus production under stress conditions.

### 3.5. Coral bleaching follows an optical positive feedback mechanism

When the ambient seawater temperature increases by 1–2°C over normal summer maximum values, corals experience thermal stress that can lead to coral bleaching via degradation or expulsion of their Symbiodiniaceae [4]. We simulated the effect of such bleaching on the coral microenvironment by running the slab model with decreasing cell density of Symbiodiniaceae in the coral tissue, as implemented in the model by decreasing the absorption coefficient of the oral and aboral gastrodermis to cover a range of corals during different stages of bleaching (2.17 × 10^6^ cells cm^−2^, 1.09 × 10^6^ cells cm^−2^, 0.54 × 10^6^ cells cm^−2^ and 0 cells cm^−2^).

**Figure 8.**
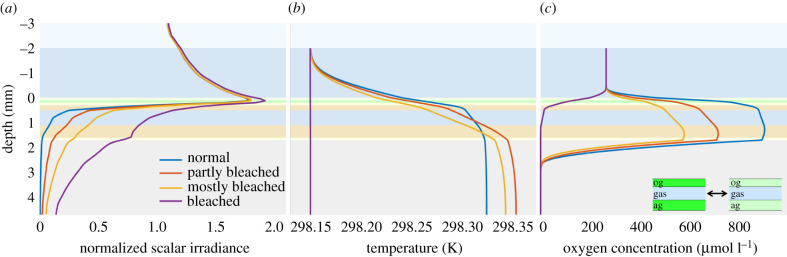
Simulated depth profiles of normalized scalar irradiance (*a*), temperature (*b*) and O_2_ concentration (*c*) for decreasing density of Symbiodiniaceae in the coral tissue during coral bleaching.

The simulated depth profiles showed drastic changes in the *in vivo* light field, temperature and O_2_ concentration in response to decreasing Symbiodiniaceae cell density (figure 8). As expected, the O_2_ concentration in the coral decreased during coral bleaching, as less photosynthetically active Symbiodiniaceae remained in the tissue. Upon complete bleaching, only the respiration of the coral host remained.

The model also indicated that light penetration into the tissue increased during coral bleaching, as less light was absorbed in the oral and aboral gastrodermis. Our simulation results are in line with experimental data obtained with bleached corals [22,70,71], where the relationship between the Symbiodiniaceae cell density and the light enhancement in the coral tissue was described by a power law (electronic supplementary material, figure S5). These observations suggest that the onset of coral bleaching results in an optical positive feedback loop accelerating high light exposure of the remaining Symbiodiniaceae as bleaching progresses.

Experiments have shown that bleached corals have lower tissue surface temperatures [12]. By contrast, our simulations of the thermal microenvironment in bleaching corals predicted an almost identical coral tissue surface temperature for all cases, while also indicating increased temperatures inside partially bleached coral tissue, especially in deeper tissues, as compared with the non-bleached coral case. The different thermal behaviour between the experiments and simulations could be explained by the way the heat source of the oral and aboral gastrodermis is modelled, which is the absorption coefficient multiplied by the local scalar irradiance. When the coral bleaches, the absorption coefficient decreases, owing to a decrease in Symbiodiniaceae cells, while the local scalar irradiance increases.

The available light component is probably larger than the absorption component, resulting in a somewhat higher temperature within the bleached corals and a similar coral tissue surface temperature to the non-bleached coral in the simulations. Only the completely bleached coral case shows no heating, since there is virtually no absorption in the coral tissue.

It is an important result that the Symbiodiniaceae cells inside the coral could experience higher temperatures than the measured coral tissue surface temperature. Thus, Symbiodiniaceae cells might actually be expelled as a function of higher tissue temperatures than the ambient water temperature, which is usually taken as a benchmark in *in situ* experiments of mass coral bleaching events [54,72]. As heat transfer and radiative heat dissipation in corals depend on external flow and tissue optics [11,12], the actual tissue temperature and thus threshold for coral bleaching is probably affected by both water flow and coral morphology, i.e. showing characteristic differences between branched and massive corals [9]. It could thus be relevant to combine this modelling approach with experimental measurements of tissue temperature and heat transfer. On the other hand, the slab model could be expanded to simulate a 3D coral microenvironment with more complex morphologies.

### 3.6. Three-dimensional hemispherical coral model

**Figure 9.**
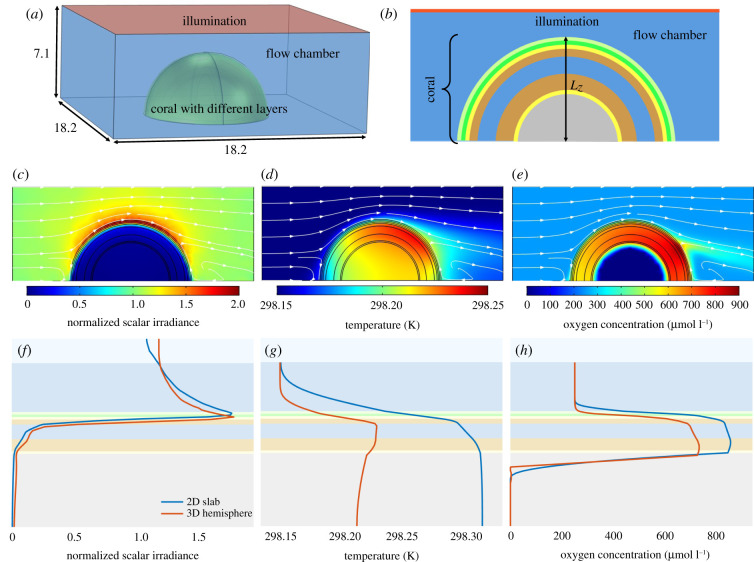
Overview and simulated profiles of normalized scalar irradiance, temperature and O_2_ concentration for a hemispherical coral model. All parameters of the 3D coral model were identical to the baseline case used in the 2D coral slab model (table 1). (*a*) Three-dimensional model geometry. The additional dimension values are in mm. (*b*) Mid-section through the hemispherical coral showing different tissue layers (see also figure 1). Two-dimensional mid-section plots of normalized scalar irradiance (*c*), temperature (*d*) and O_2_ concentration (*e*), with the flow indicated by white arrowed streamlines. (*f*–*h*) Depth profiles comparing the 3D hemispherical and 2D slab coral models for the baseline case. The 3D data were sectioned along a vertical line through the centre of the hemispherical coral.

Our simple coral slab model could simulate various real-life scenarios of coral microenvironments that qualitatively matched actual published measurements of the optical, thermal and chemical microenvironment in corals surprisingly well. Using such a computationally simple model could thus be a useful tool for simulating and thereby predicting the microenvironment in corals with specific tissue and skeleton layers and for different light and flow scenarios. However, we are well aware that this approach is a simplification of the more complex 3D structure of corals, which could lead to important changes in the radiative, heat and mass transfer-derived physical and chemical microenvironment. To provide an outlook and stepping stone for future 3D coral models, the same baseline case was modelled for a 3D hemispherical coral (figure 9). All parameters were kept the same as in the 2D coral slab model, while the different tissue layers are represented as hemispherical shells in the 3D coral. The 3D hemispherical shape of the coral caused self-shading of both sides corresponding to the top illumination (figure 9*c*), and the effect of flow over the hemispherical coral model led to a skewed distribution of temperature and O_2_ concentration as a result of flow recirculation (figure 9*d,e*).

The depth profiles of scalar irradiance, temperature and O_2_ concentration along with a vertical transect through the centre of the hemisphere exhibited a similar shape to that in the 2D slab model (figure 9*f*–*h*). As seen from figure 9*f*, the irradiance in the 2D slab model and the 3D hemisphere model is very similar along with the vertical line profile taken in the middle of the geometry. The differences observed in the temperature and O_2_ microprofiles are due to at least three effects. First, the flow profiles in the 3D and 2D simulations are not the same, which results in different TBL and DBL thicknesses. That is, the TBL and DBL in three dimensions are thinner, which leads to decreased temperatures and O_2_ concentrations in the coral tissue because of more efficient heat and mass transfer. Second, the third spatial dimension results in added dissipation of heat and mass, which further decreases the temperature and O_2_ concentration. Finally, irradiance reduces away from the centre (figure 9*c*—2D mid-section irradiance plot) because of an increasing incidence angle of light, owing to the slant surface of the 3D structure. This increases back reflections at interfaces with refractive index mismatch, causing more loss of light and hence lower production of oxygen and heat.

## 4. Summary and outlook

We developed a simple coral slab model that enables simulations of how coral microenvironments are linked to tissue structure and composition under experimental conditions typically used for microscale analyses of corals in the laboratory. The model is mostly limited by the light field simulations and the simplified geometry and flow scenario.

The 3D MC simulations were performed with photons of one wavelength (636 nm) overlapping with the major absorption window of the coral microalgal symbionts containing Chl*c* and Chl*a*, taking only scattering and absorption into account and ignoring fluorescence. Clearly, coral optics and photobiology involve both UV and solar radiation in the full visible and near-infrared spectrum [3], and our model so far does not include, for example, the effects of fluorescent coral host pigments changing harmful UV and blue wavelengths towards less harmful red-shifted light [73], and possible positive effects of such shielding and wavelength transformation processes on coral photosynthesis [16,74]. Furthermore, the absorption of more energy-rich radiation in the UV-blue spectral range may lead to more heat dissipation and thus higher temperatures in coral tissue [12] than predicted in our present model. Accounting for such spectral interactions could, for example, build on existing models developed in biomedical optics that include fluorescence in radiative transfer modelling [75,76], but they would need more computationally intensive MC simulations, more detailed spectral information on the inherent optical properties of coral tissue layers and skeleton, as well as the fine-scale distribution and density of algal symbiont and host pigments in coral tissue (e.g. [26]).

While the implementation of a 2D slab geometry in our coral model could be used to verify basic mechanisms affecting the physico-chemical microenvironment in corals by comparing model outputs with published experimental data from flow chamber studies, it does certainly not capture every component of coral optics and biology because of the strong simplification of geometry and the hydrodynamic regime. For example, in a more complex (3D) model, differences in coral morphology could be included that have an impact on the water flow and on the temperature of corals [9,32]. Furthermore, the underlying seabed could be included in the coral optics, since it can contribute by over 20% to the total scalar irradiance at the coral tissue surface in shallow waters [37].

Our 2D representation of a coral slab is in principle aligned with the way microsensor measurements in corals are commonly interpreted—that is, an essential 1D description of photon, heat and mass transfer between water, tissue and skeleton ignoring geometry effects (e.g. [9,13,19]). However, coral geometry (at both the single polyp and colony scale) and its interaction with the flowing seawater is important for the coral microenvironment. While a faster flow can enhance mass and heat transfer around coral structures (ridges and branched tips), low flow, e.g. around retracted polyps and in between coral branches/ridges, can lead to enlargements of thermal and diffusive boundary layers and local point measurements being increasingly affected by the surrounding coral topography (e.g. [11,13,77,78]). Local enhancements of mass transfer due to ciliary movement can also affect the chemical microenvironment at the coral polyp scale (e.g. [79]); at a larger colony scale, the complex interaction of turbulent flow with 3D coral morphology leads to a heterogeneous distribution of temperature and O_2_ (e.g. [32,80]). Additionally, photon transfer is affected by coral topography, where, for example, skeleton morphology and optical properties can affect the distribution of light within the coral colony [28,31,60,81]. So, although our model apparently can simulate the local coral tissue microenvironment as measured in several published microsensor studies, it is important to now incorporate 3D coral morphology and more complex flow scenarios to simulate the spatial heterogeneity of microenvironments over coral colonies.

A more elaborate 3D model would enable more realistic simulations of the role of tissue and skeleton morphology in different coral species, but it would still rely on more accurate estimates of the optical properties for the different coral tissue and skeleton layers. Some advancements have been made in the techniques for measuring bulk coral tissue and skeleton optical properties [26,37]; however, further technical improvement is required to measure optical properties of specific coral tissue layers [25]. Last not least, our model currently includes a very simple representation of coral and Symbiodiniaceae metabolism, where we estimate photosynthesis and respiration rates from published values of photosynthesis efficiency and photosynthesis : respiration ratios in corals, e.g. ignoring any light saturation or photoinhibition. The integration of more detailed metabolic pathways and functions (e.g. nutrient and carbon flow, substrate degradation pathways or oxidative stress) in the different compartments of the coral holobiont, i.e. the coral host animal with its algal symbionts and associated microbiomes, would enable a more comprehensive exploration of interactions between coral metabolism and the physico-chemical microenvironment.

In summary, we developed a multiphysics model linking radiative transfer, heat and mass transfer in a simple slab model representative of a flow- and light-exposed coral with different tissue layer and skeleton compartments. This enabled us to simulate the effect of different tissue characteristics (i.e. tissue thickness, tissue movement, density of Symbiodiniaceae, density of coral host pigments as well as mucus secretion) on the coral microenvironment and metabolism (in terms of photosynthesis and respiration). While admittedly being a simple 2D representation of complex coral morphology, our model and the simulated scenarios provided insight into several key mechanisms affecting the coral microenvironment, and the light, temperature and O_2_ profiles predicted by our simulations were generally very consistent (in both shape and magnitude) with published experimental data on the coral microenvironment (based either on a coral tissue surface or on profile measurements of corals maintained in simple flow chambers). Our coral model also predicted within-tissue and skeleton dynamics of the physico-chemical microenvironment (e.g. in terms of tissue temperature dynamics during coral bleaching) that await experimental testing—to the extent that existing microsensor and imaging technologies can resolve light, temperature and O_2_ gradients at the necessary resolution.

## Data accessibility

The Matlab codes and data are provided in the electronic supplementary material [82].
